# Nanocomposite films as electrochemical sensors for detection of catalase activity

**DOI:** 10.3389/fmolb.2022.972008

**Published:** 2022-09-26

**Authors:** Dwight Johnson, Unyoung Kim, Maryam Mobed-Miremadi

**Affiliations:** Biological Micro/Nanosystems and Biomaterials Engineering Laboratories, Department of Bioengineering, Santa Clara University, Santa Clara, CA, United States

**Keywords:** biosensor, electrochemical, alginate, chitosan, CNT, nanocomposite, catalase, encapsulation

## Abstract

Cross-linked hydrogel substrates have garnered attention as they simultaneously enable oxidoreductase reactions in a control volume extended to adsorption of redox capacitors for amplification of electrochemical signals. In this study, the effect of catalase immobilization in mold-casted alginate-based thin films (1 mm × 6 mm × 10 mm) containing multi walled carbon nanotubes (MWCNT) coated with chitosan has been studied via amperometry. The amperometric response was measured as a function of peroxide concentration, at a fixed potential of −0.4 V vs. SPCE in phosphate-buffered saline (pH = 7.4). Results indicate substrate detection is not diffusion-limited by the 100 μm thick chitosan layer, if the cationic polyelectrolyte is in contact with the sensing carbon electrode, and the linear detection of the enzyme absent in solution is enabled by immobilization (*R* ^2^ = 0.9615). The ferricyanide-mediated biosensor exhibited a sensitivity of 4.55 μA/mM for the optimal formulation at room temperature comparable to other nanomaterial hybrid sensing solution namely amine-functionalized graphene with an average response time of 5 s for the optimal formulation. The suitability of the optimized chitosan-coated alginate slabs nano-environment for co-encapsulation of catalase and carbon nanotubes was confirmed by cyclic voltammetry.

## 1 Introduction

Alginate is one of the most abundant anionic biopolymers first extracted from marine algae and subsequently isolated from differentiated bacteria (Slack and Nichols, 1981; Nordgård and Draget, 2021). Due to this fact, its biocompatibility (Wong and Chang, 1991; Lee and Mooney, 2012; Bochenek et al., 2018) and biodegradability the applications span the food, climate and health nexus (Shaari and Kamarudin, 2015; Nesic and Seslija, 2017; Gao et al., 2020; Gheorghita Puscaselu et al., 2020). Due to bioresorbable properties under physiological conditions, alginate is the most assayed polyelectrolyte in hard and soft tissue engineering where bioerosion is regulated by crosslinking the polymer into networks using a multitude of bio-fabrication methods (Matricardi et al., 2013; Kim and Kim, 2015). Enhancement strategies for regulating the mechanical and conductive properties include the incorporation of several amounts of carbon nanomaterials (CNMs), such as carbon nanofibers (CNFs), graphene oxide (GO) (Llorens-Gámez and Serrano-Aroca, 2018; Serrano-Aroca et al., 2018; Llorens-Gámez et al., 2020; Hurtado et al., 2022), and other graphene-based materials have been proposed (Ahadian et al., 2014; Golafshan et al., 2017; Liu et al., 2018; Fu et al., 2019; Li et al., 2019). Chitosan, a polycationic, bacteriostatic biopolymer extracted from crustaceous shells (Croisier and Jérôme, 2013; Goy et al., 2016), is widely used with alginate as a coating applied by physical adsorption (Abbaszadeh et al., 2014) across the above-mentioned applications. Chitosan is used in polymer blends as a filler to improve the dielectric properties of the mixture (Johns and Nakason, 2011; Bonardd et al., 2018) and in hydrogels nanocomposites as a biomimetic conductor (Croisier and Jérôme, 2013; Mihic et al., 2015; Cui et al., 2018).

The development of conductive hydrogels comprised of alginate, chitosan and carbon nanotubes has great potential in cutting edge bioelectronics that allow the application of electrostimulation, smart bandages and stretchable electronics as healthcare converging towards telemedicine and decentralized trials (Lin et al., 2016; Gilshteyn et al., 2019; Yuk et al., 2019; Derakhshandeh et al., 2020). Among these developments, physiological measurements can be used to determine different types of responses in the body namely reactive oxygen species (ROS) that are generated in cells as endpoints during injury and wound healing when basal concentrations of hydrogen peroxide (H_2_O_2_) shed light on the different stages. Elevated levels of hydrogen peroxide are therefore a biomarker of possible infection (Serra et al., 2008; Robinson, 2022; Lipcsey et al., 2022). The use of synergetic effects of oxidoreductases namely fungal Manganese Peroxidase, horseperoxidase (HRP) and catalase (CAT) mixed with a cocktail of other enzymes has also been investigated in bioremediation by means of lignolytic enzyme activity, a specific example being the environmental friendly production of 2,5-Furandicarboxylic acid (FDCA), one of the top lignocellulosic-derived value-added chemicals as a substitute to petroleum-based plastics (Babič et al., 2012; Cajnko et al., 2020).

**FIGURE 1:**
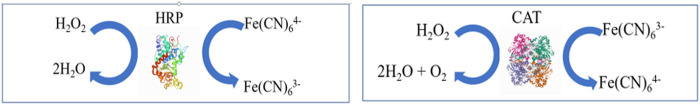
Redox reactions and associated mediators for the electrochemical detection of catalase **(A)** (left) Oxidation of hexacyanoferrate (II) by HRP, **(B)** (right) reduction of hexacyanoferrate (III) by H_2_O_2_. Molecular structure of the enzymes obtained from the protein database (Berglund et al., 2022; Sugadev et al., 2022).

Among the ROS products superoxide (O_2_^−^) is formed as a direct result of cellular metabolism the concentration of which is regulated by superoxide dismutase converting the substrate into oxygen (O_2_) and hydrogen peroxide. The peroxide can be oxidized by HRP or reduced by CAT. *In vitro*, the overpotential generated by the electrochemical reaction can be reduced by use of the potassium hexacyanoferrate (II)/(III) complexes for which the direction of the equilibrium (Dunford and Hasinoff, 1970; Berglund et al., 2002; Berlung et al., 2002; Sitnikova et al., 2014; Berglund et al., 2022; Sugadev et al., 2022) as illustrated in Figure 1.

Many studies have investigated immobilization of catalase on sensor surfaces in order to promote electrocatalytic activity. Catalase immobilized on nanocomposite modified electrodes exhibit high sensitivity, low detection limit and improved catalytic activity (Schubert et al., 1991; Li et al., 1996; Chen et al., 2007; Prakash et al., 2009; Hong et al., 2013). Many methods have been explored to detect hydrogen peroxide, such as UV-vis spectrophotometry, titrimetry, chemiluminescence and electrochemistry to name a few (Cui et al., 2007; Huang et al., 2011; Woo et al., 2012; Pundir et al., 2018; Soto et al., 2021; Ahmad et al., 2022). Various electrochemical biosensors for H_2_O_2_ sensing have been explored by immobilizing protein and enzyme in different materials such as immobilization of multi-wall carbon nanotubes (CNT) (Salimi et al., 2005), catalase with single-walled carbon nanotubes-chitosan (Jiang et al., 2008), catalase (CAT) with amine-functionalized graphene (graphene-NH_2_) and gold nanoparticles (AuNPs) (Huang et al., 2011), iron nanoparticles with graphene’s layers on multi-wall carbon nanotubes (Cui et al., 2007), enzymes immobilized on poly (glycidyl methacrylate-co-vinylferrocene) (Şenel et al., 2010; Şenel et al., 2011). Toward the goal of developing a highly sensitive catalase-based sensor for hydrogen peroxide detection, a screening of driving forces comprised of chitosan conductivity and immobilization kinetics was conducted to derive the optimal composition and stacking order of the nanocomposite thin films based on amperometric detection sensitivity.

## 2 Materials and methods

### 2.1 Materials

The following chemicals and materials used to make the sensors were purchased from Millipore-Sigma (Burlington, MA) low-molecular-weight chitosan (44,886–9, 75% deacetylated, 3.8–6.0 kDa), alginic acid sodium salt (71,238), catalase (C40, ≥10,000 units/*mg* protein, MW = 250 kDa), horseradish peroxidase (P8250, 150–250 units/*mg* protein, MW = 44 kDa), carbon nanotubes (698,849, multi-walled,>98% carbon basis, O.D. x L 6–13 nm x 2.5–20 μm). Potassium ferricyanide (P232500), potassium ferrocyanide trihydrate (P236500) and hydrogen peroxide (H312-500) as well as all other reagent grade salts, solvents, were procured from Fisher Scientific (Pittsburgh, PA). Screen-printed electrochemical sensors (DRP 110) and the boxed connector for the sensors (DRP-DSC) were purchased from Metrohm Dropsens (Oviedo, Spain). The electrochemical sensors consist of carbon working, silver reference, carbon counter electrodes abbreviated as SPCE.

### 2.2 Preparation of nanocomposite sensing platform

#### 2.2.1 Composition

Components of nanocomposite gels and respective controls (w/wo enzyme, w/wo CNT) were prepared according to the following steps. Carbon nanotube powder (CNT) at the concentration of (0.12 mg/ml) was added to 3.0% sodium-alginate (A) solution dissolved in 0.9% (w/v) NaCl (saline) followed by vigorous mixing for 1 h. Horseradish peroxidase (HRP) or catalase (CAT) were added to the blend and allowed to mix for an additional 30 min for final concentrations of 5 mg/ml (114 μm) and 1 mg/ml (4 mM), respectively. Hydrogel shrinkage estimated at 30% was measured volumetrically to determine the final concentrations of enzyme and CNT. Chitosan was dissolved at 1% (*w/v*) in a sodium acetate buffer solution (pH = 4).

#### 2.2.2 Biosensor fabrication

**FIGURE 2:**
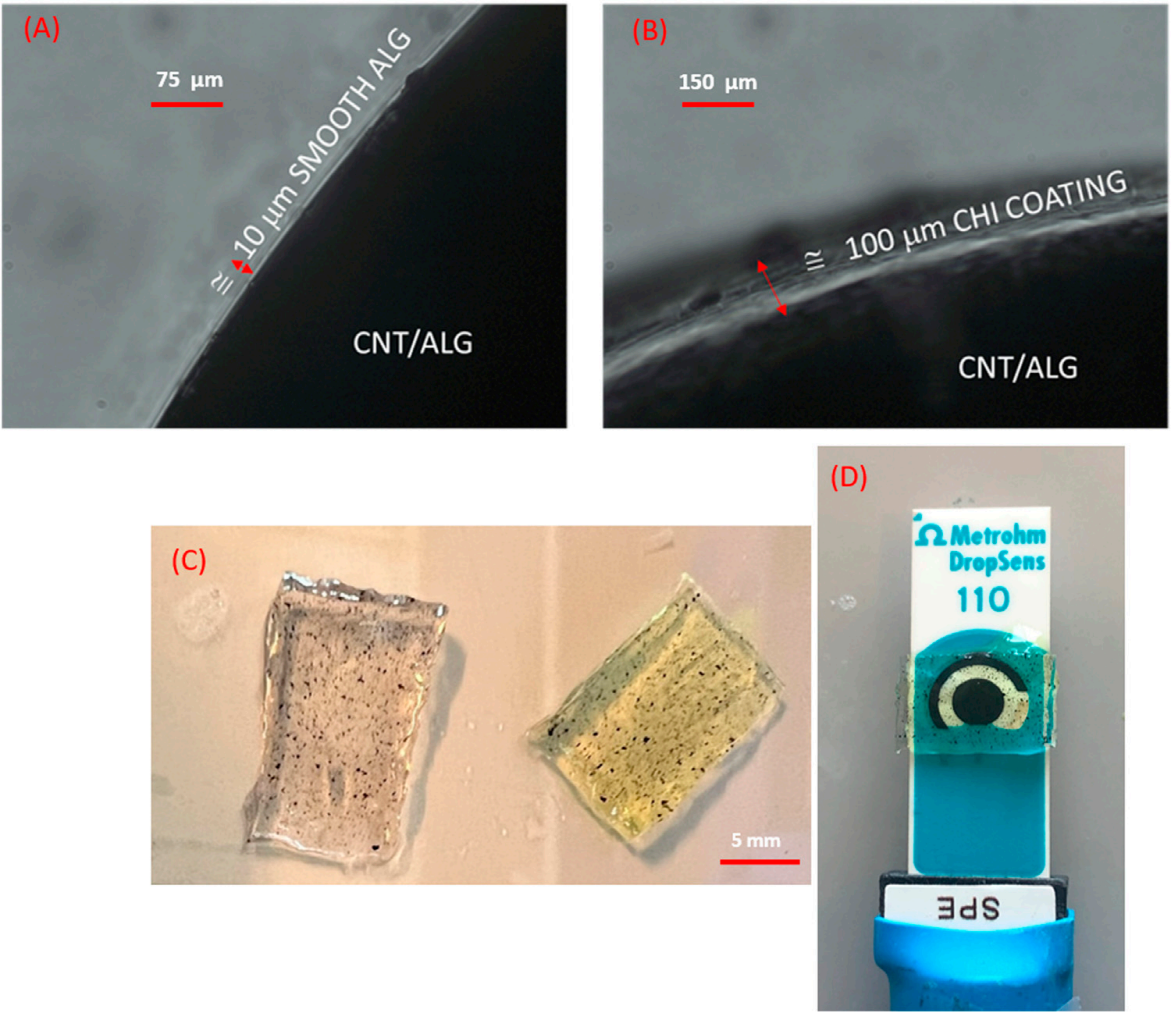
Surface coating of nanocomposites **(A)** Alginate control film containing SWCNT, **(B)** Chitosan coated alginate film containing SWCNT with a 100 μm added thickness captured at ×40 magnification; **(C)** Nanocomposite film prior (translucent) and post (translucent green) electrochemical processing; **(D)** Screen-printed sensor with carbon working, silver reference, carbon counter electrodes and nanocomposite film used for H_2_O_2._

Alginate slabs and respective controls (w/wo enzyme, w/wo CNT) averaging 1 mm in thickness (area ≅ 10 mm × 6 mm) were fabricated by polyelectrolyte complexation of alginate after incubation into a 1.5% (*w/v*) of CaCl_2_ for 20 min followed by saline washes using a previously established method (Mobed-Miremadi et al., 2013a). A subset of the mold-casted films were immersed into a chitosan bath for 20 min to conduct the coating step by physical adsorption enabled by the electrostatic attraction of the amine groups to the alginic acid. Successful adsorption was verified using microscopy (Leica model # DMI3000 B, Wetzlar, Germany) where the coating adds approximately 100 μm to the thickness of the slabs as presented in Figure 2. CNT incorporation was measured using visual inspection at 1X. The dimensions of the slab were measured using a caliper (Mitutoyo model #500–196–30, Kawasaki, Japan). The sensors were left in PBS until testing to avoid shrinkage. Approximately, 60 s before testing hydrogel films transferred into H_2_O_2_ bath of varying concentration in order to reduce diffusion limitations.

### 2.3 Electrochemical testing setup

The SPCE sensor was connected to an electrochemical analyzer (CH Instruments, Austin, TX). Measurements were conducted at a pH of 7.4 and room temperature ranging between- 20–25°C. Presented in Table 1 are sensor composition, stacking order with respect to SPCE contact, and associated nomenclature.

**TABLE 1:** Sensor composition and stacking order with respected to contact with SPCE.

*Amperometry*			*Voltammetry*		
Nomenclature	Number of polymer layers	Layer in contact with SPCE	Nomenclature	Number of polymer layers	Layer in contact with SPCE
Free HRP	N/A	H2O2 + HRP + Ferro	A	1	A
RSHRP	2	A/CNT + HRP (*RS*)	AE	1	A/CAT
SHRP	2	Chi (*S*)	A/CNT	1	A/CNT
Free CAT	N/A	H2O2 + CAT + Ferri	A/CNT + E	1	A/CNT + CAT
RSCAT	2	A/CNT + CAT (*RS*)	A/CNT/Chi	2	Chi (*S*)
SCAT	2	Chi (*S*)	A/CNT/Chi + E	2	Chi (*S*)

### 2.3.1 Amperometry

For detection of HRP and catalase activity amperometry was performed in the range of 0.1 V to −0.4 V. For each test a new electrochemical sensor was used.

For detection of enzyme activity in solution, a 10 μl solution of HRP (5 mg/ml = 4 μm) or CAT (1 mg/ml = 114 μm) was added onto the sensor surface followed by 20 μL of mediator solution (50 mM hexacyanoferrate (II) (Ferro) or hexacyanoferrate (III) (Ferri) in 0.1 M PBS, pH 7.0) to which 20 μL of 0.1 M PBS was added to make a final volume of 50 μL. The reaction was initiated by adding a 200 mM H_2_O_2_ of solution at an increment of 1 μL until an upper substrate concentration of 3 μL was reached. For detection of enzymes immobilized in nanocomposite slabs, 20 μL of mediator was added, followed by addition of 30 μL of 0.1 M PBS for deposition onto the sensor followed by incremental substrate addition as described above.

The signal to noise ratio (*S/N*) given by Eq. 1, defined as the ratio of the maximum current at a given substrate concentration (*I*_max*@S*_) by the maximum current in the absence of substrate (*I*_max*@S=0*_) at a given voltage was used to evaluate optimal voltage for kinetic evaluations.

(SN)=Imax⁡@SImax⁡@S=0 (1)

The response time corresponding to the steady-state current (*I*_*SS*_) across scanned voltages per immobilization state was recorded.

### Cyclic voltammetry

Cyclic voltammetry was only performed on nanocomposite slabs with a voltage sweep between 0.5 V and −0.8 V at a rate of 100 mV/s. Peak current was observed at −0.4 V where the maximum (S/N) was registered for catalase. Specifically, the slope from −0.6 V to −0.2 V was constructed and subsequently the corresponding peak current was measured and reflected in plots. A single sensor was used for all comparisons. Similar to amperometry, 20 μL of mediator was added, followed by addition of 30 μL of 0.1 M PBS to make a final volume of 50 μL mixture for deposition onto the sensor. The H_2_O_2_ substrate at a concentration of 200 mM was added at an increment of 1 μL until an upper substrate concentration of 3 μL was reached.

## 3 Results and discussion

### 3.1 Amperometric detection of HRP and catalase

**FIGURE 3:**
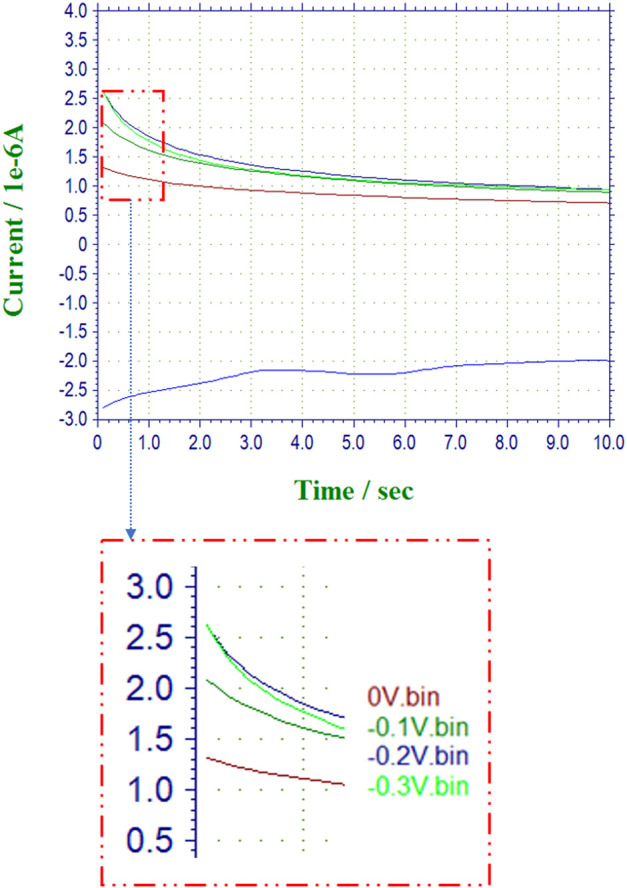
**(A)** (top) Superimposed sample amperograms for Free HRP and 8 mM of H_2_O_2_ recorded at multiple voltages for a total scan time of 10 s using which the steady state sampling times were estimated. **(B)** (bottom) Outset dashed magnified region for the determination of the maximum current (I_max@S_) used for the signal to noise estimations.

**FIGURE 4:**
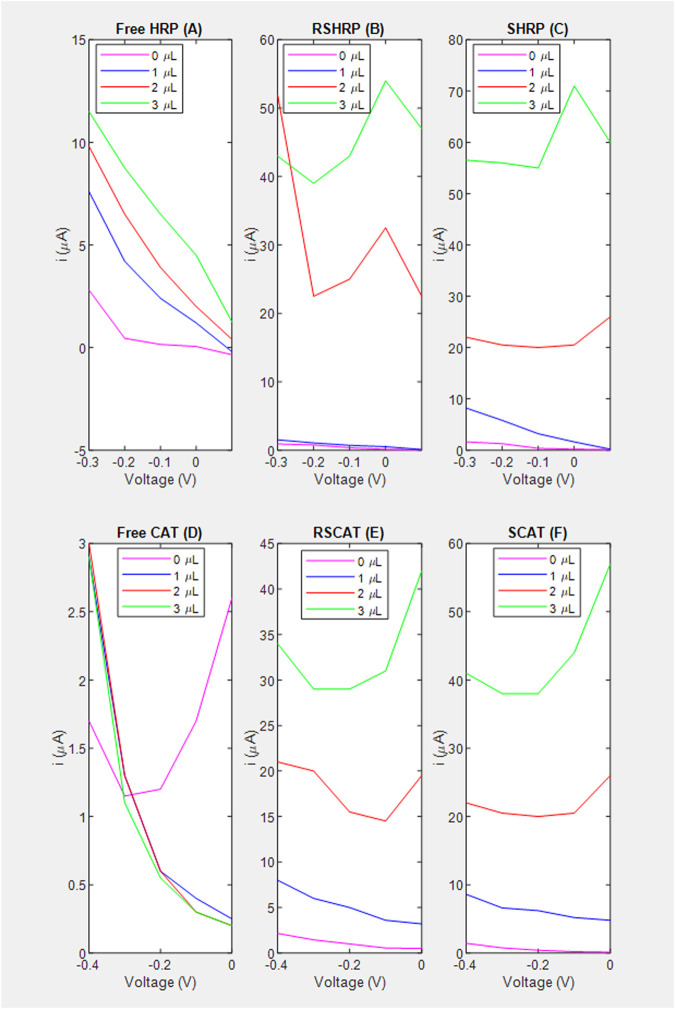
Voltage vs. current based on amperometric data for the estimation of the signal to noise ratio. **(A)** Free enzyme; **(B)** RSHRP nanocomposite film where chitosan is not in contact with the SPCE; **(C)** SHRP nanocomposite film where chitosan is in contact with the SPCE; **(D)** Free enzyme denoted as FreeCAT; **(E)** RSCAT nanocomposite film where chitosan is not in contact with the SPCE; **(F)** SCAT nanocomposite film where chitosan is in contact with the SPCE.

Shown in Figure 3 is a sample amperogram for HRP captured at multiple voltages for the lowest substrate concentration of 4 *mM* for the determination of the maximum current (*I*_max*@S*_). The signal to noise ratios for the multiple configurations of the nanocomposite sensors as compared to the free enzymes are presented in Table 2 and Figures 4A–E. The voltage at which the (S/N) ratio was highest for the free enzyme at the lowest substrate concentration tested (4 mM = 1 μL of H_2_O_2_) was chosen for reporting the kinetic activities. The voltage chosen for catalase is −0.4 V in agreement with literature (Prakash et al., 2009). The highest signal intensity with reference to the background noise was recorded at 0 V, however a voltage of −0.1 V was chosen for comparative purposes to previously reported findings (Sekar et al., 2015). This low potential for H_2_O_2_ sensing is preferable as it minimizes potential interferences compared to direct oxidation of H_2_O_2_ near 0.7 V vs. SCE.

**TABLE 2:** Signal-to-Noise values for amperometric measurements.

	Voltage (*V*)	FREEHRP	SHRP	RSHRP	Voltage (V)	FREECAT	SCAT	RSCAT
1 μL [H_2_O_2_] = 4 mM	0	24	9	2	-0.1	0.24	26	6.5
	−0.1	16	5	1	-0.2	0.50	16	5.0
	−0.2	9.3	5	2	-0.3	1.1	9	4.1
	−0.3	2.7	5	2	-0.4	1.7	6	3.7
2 μL [H_2_O_2_] = 8 mM	0	40	77	71	-0.1	0.18	100	26
	−0.1	26	24	30	-0.2	0.5	50	16
	−0.2	14	22	58	-0.3	1.1	27	14
	−0.3	3.5	18	21	-0.4	1.8	15	10
3 μL [H_2_O_2_] = 12 mM	0	90	160	120	-0.1	0.18	220	56
	−0.1	43	45	52	-0.2	0.46	95	29
	−0.2	19	35	48	-0.3	0.96	51	20
	−0.3	4.1	28	34	-0.4	1.7	29	16

#### Effect of chitosan conductivity and nanocomposite layer stacking order

**FIGURE 5:**
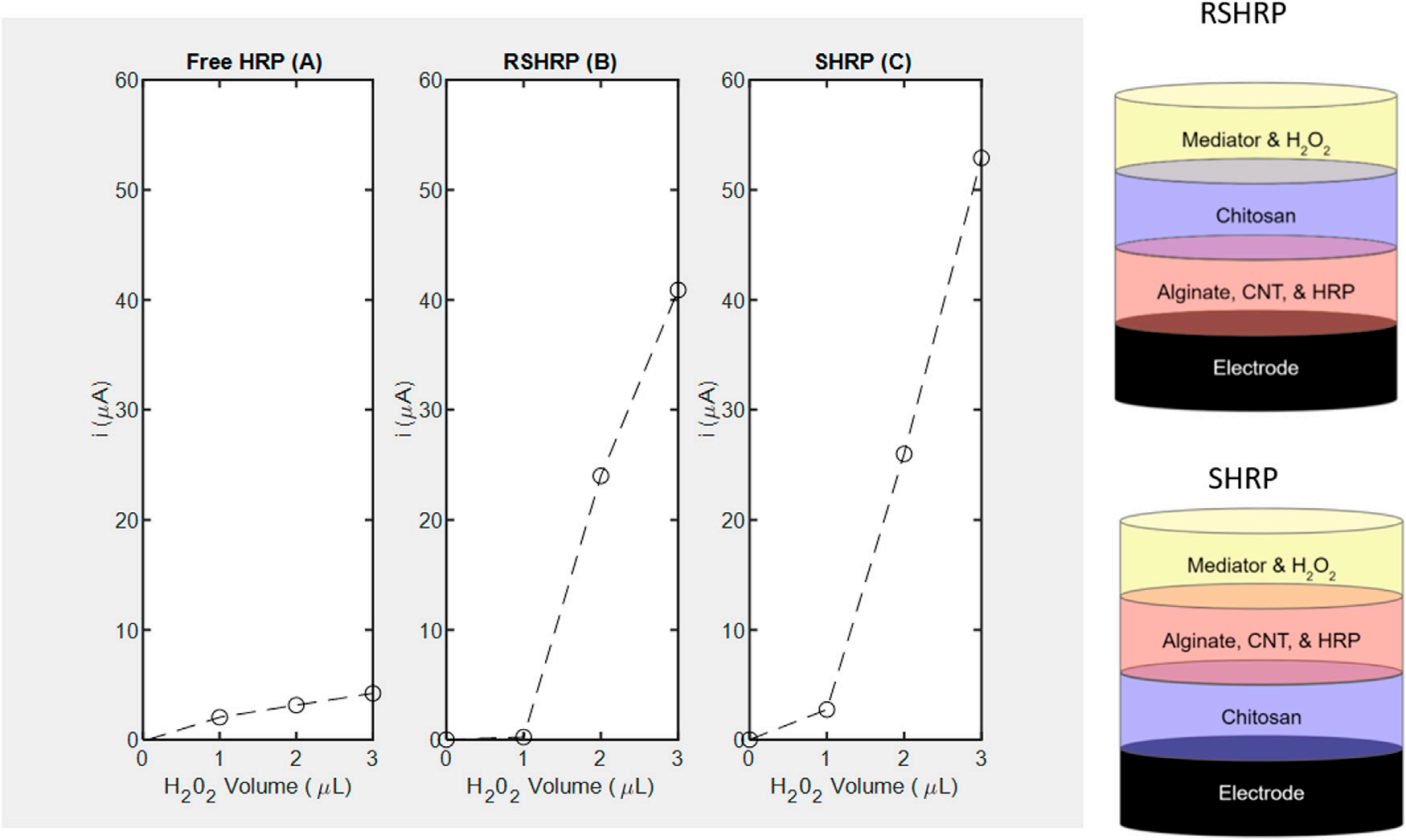
Amperometric studies of horseradish peroxidase measured at −0.1 V using H_2_O_2_ as substrate and corresponding layer stacking configurations (not drawn to scale). **(A)** Free enzyme; **(B)** RSHRP nanocomposite film where chitosan is not in contact with the SPCE; **(C)** SHRP nanocomposite film where chitosan is in contact with the SPCE.

**FIGURE 6:**
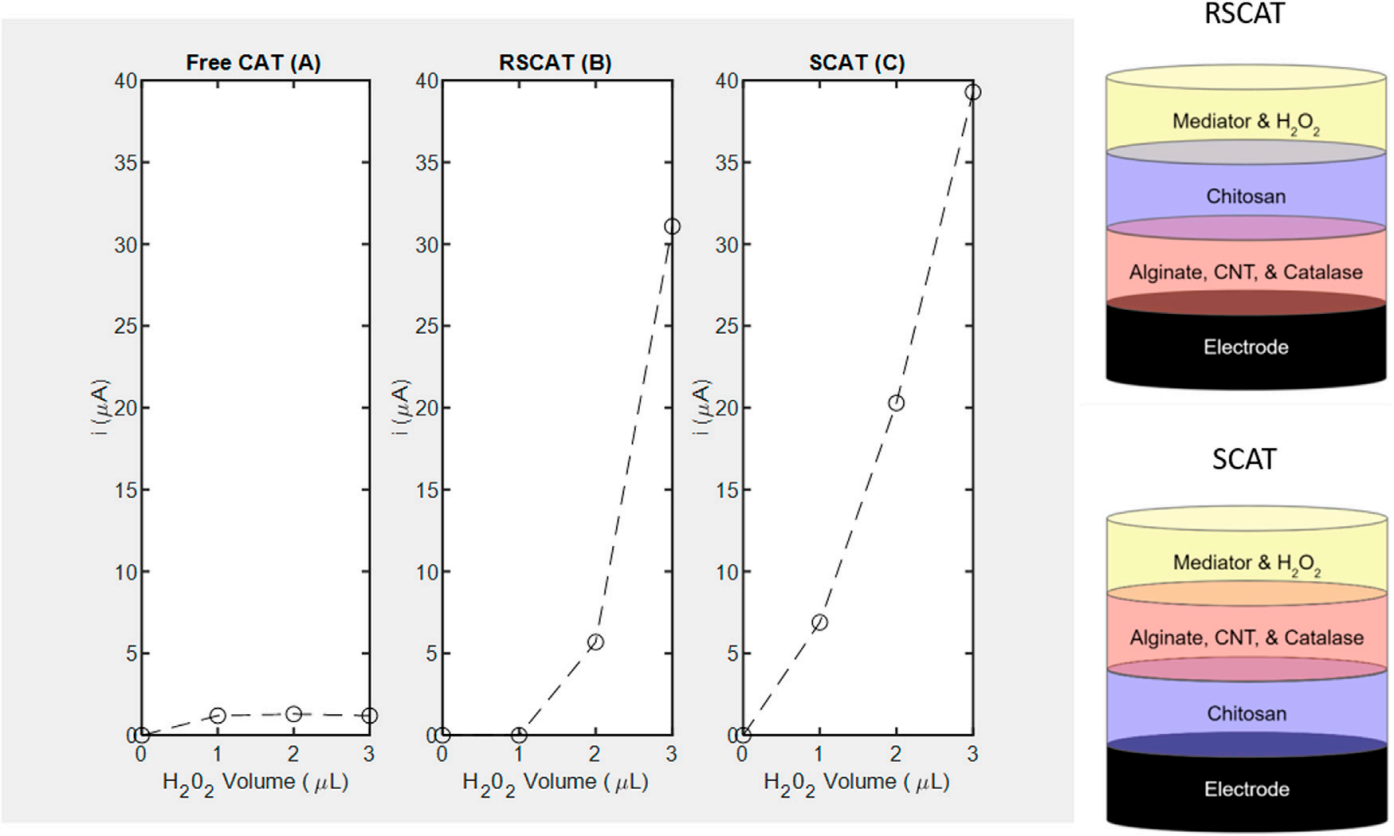
Amperometric studies of catalase measured at -0.4 *V* using H_2_O_2_ as substrate and corresponding layer stacking configurations (not drawn to scale). **(A)** Free enzyme denoted as FreeCAT; **(B)** RSCAT nanocomposite film where chitosan is not in contact with the SPCE; **(C)** SCAT nanocomposite film where chitosan is in contact with the SPCE.

A common observation for both enzymes is that chitosan contact with the electrode (S stacking configuration) improves the lower limit of detection (LOD) of H_2_O_2_ to 1 *μL* equivalent to 4 *mM* for the nanocomposite films (Figures 5B vs. Figure 5C; Figures 6B vs. Figure 6C). For HRP, implementation of the immobilization scheme (Figures 5C vs. 5A) does not improve the lower limit of detection (LOD), however the sensitivity ratio calculated based on the slopes of SHRP (6.27 μA/mM) vs. Free HRP (0.3545 μA/mM) above the LOD was determined to be 17.0. The calibration curve for the free enzyme is linear (*R* ^2^ = 0.9656) with an LOD of 1 μL.

As for CAT, immobilization in a specific geometric orientation where the chitosan is in contact with the electrode (SCAT) enables the LOD of 4 mm (Figure 6C) absent in the non-linear free enzyme signal (*R* ^2^ = 0.5965) presented in Figure 4A. The sensitivity for the SCAT sample has been determined to be 4.55 μA/mM) characterized by a coefficient of determination of 0.9615. Table 3 summarizes the recent study of nanocomposite-based H_2_O_2_ sensors, and the calculated sensitivity from this study performs within the range without the need of functionalizing the electrode nor cross-linking the enzyme (Salimi et al., 2005; Cui et al., 2007; Jiang et al., 2008; Hong et al., 2013; Pundir et al., 2018; Ahmad et al., 2022).

**TABLE 3:** Sensitivity of hydrogen-peroxide nanosensors.

Authors	Electrode composition	Sensitivity (μA/mM)	Response time (s)
K.J. Huang et al.^50^	CAT/graphene-NH_2_ and AuNPs	13.4	2
D. Soto et al.^51^	Fe/graphene/CNTs	7.41	Not reported
A. Salimi et al.^55^	MWCNTs/GCE	3.30	<2
H.J. Jiang et al.^56^	CAT/SWNTs-Chi/GCE	6.30	Not reported
M. Şenel et al.^57^	CAT/(poly (GMA-co-VFc))	1e-3	<7
M. Şenel et al.^58^	HRP/(poly (GMA-co-VFc))	7.36e-4	<4
Current Finding	SCAT/Amperometry	4.55	<5
Current Finding	SHRP/Amperometry	6.27	<5

#### Effect of immobilization on reaction kinetics

For the free enzymes, the discrepancy in the linear detection behavior could be attributed to the ratio of Stokes’ radii, where catalase (r_CAT_ = 4.6 nm) is twice the size of horseradish peroxidase (r_HRP_ = 2.5 nm) (Fournier, 2012). Catalase overcrowding at the electrode may have contributed to diffusion limitations at higher substrate concentrations resulting in lack of linearity for the free HRP although experimental provisions were taken to set the ratio of the enzyme concentrations 5:1 (HRP:CAT).

The twofold advantages of a control volume enabled by immobilization result in signal amplification at higher substrate concentrations. The reactions are not diffusion-limited and the chitosan enhances the conductivity of the nanocomposite layer when in contact with the carbon electrode. In the stacking order referred to as reverse sandwich (RS), chitosan acts as a diffusion barrier at lowest concentration of 4 *mM* of H_2_O_2_.

With regards to the differences in computed sensitivities in the optimal stacking configuration specifically for SHRP and SCAT, the molecular weight cutoff of the nanocomposite membrane established by multiple sources to be 3 nm (Mobed-Miremadi et al., 2013b) may suggest two different kinetic mechanisms for the ROS enzymes. While the HRP may diffuse out of the membrane for a surface reaction to occur, CAT kinetics are driven by substrate diffusion. The response time to reach the steady state current was less than 5 s across enzyme immobilization states for both enzymes based on amperometric curves.

Other means to deconvolute kinetic and diffusing driving forces would be to control the reaction temperature. The dependence of amperometric current on temperature in an initial region can be expressed as an Arrhenius relationship (Thirsk and Harrison, 1972; Wu et al., 2017). In alignment with the optimal catalysis temperature under physiological conditions of 37°C, amperometric studies of catalase and horseradish peroxidase immobilized on poly (glycidyl methacrylate-co-vinylferrocene) for H_2_O_2_ detection have been characterized by optimal kinetic performance between 40–45*°C* (Şenel et al., 2010; Şenel et al., 2011) for a pH range between 7.0–7.4 comparable to the current study.

In order to further establish the suitability of the catalase biosensor in the presence of diffusion limitations and confirm the optimal geometry and composition of the sensor platform, cyclic voltammetry was conducted using additional controls. When the adsorbed chitosan was the constituent of the sensor films, the experiments were conducted in the S configuration where the electrode comes into contact with the cationic polyelectrolyte.

### 3.2 Cyclic voltammetry of catalase activity

Applied voltage is critical in the response of mediated biosensing applications (Tatka and Kim, 2016; Wu et al., 2017). To that effect cyclic voltammetric response of the above sensors was measured in the range of 0.5 V to −0.8 V at a rate of 0.1 V/s.

#### Effect of various nanomaterial composites in the electrochemical response

**FIGURE 7:**
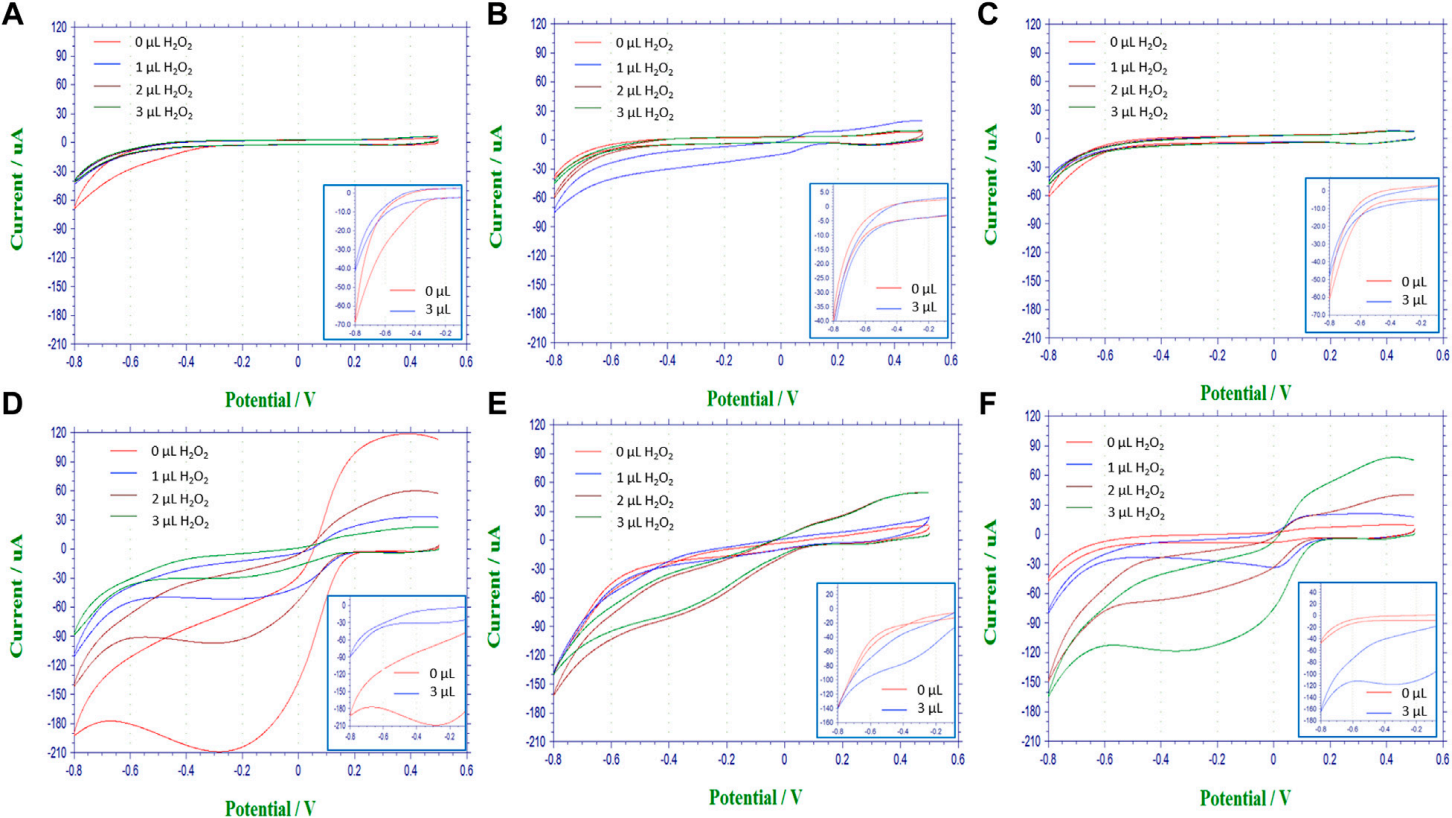
Voltammograms of nanocomposite biosensing films performed with a voltage sweep between 0.5 V and −0.8 V at a rate of 100 mV/s on controls and corresponding reactive catalase immobilized slabs in the top and bottom rows, respectively. **(A)** alginate slabs; **(B)** alginate slabs with CNTs (A/CNT); **(C)** alginate slabs with CNTs and chitosan (A/CNT/Chi); **(D)** alginate slabs with CAT (AE); **(E)** alginate slabs with CNTs and CAT (A/CNT + E); **(F)** alginate slabs with CNTs, chitosan and CAT (A/CNT/Chi + E).

**FIGURE 8:**
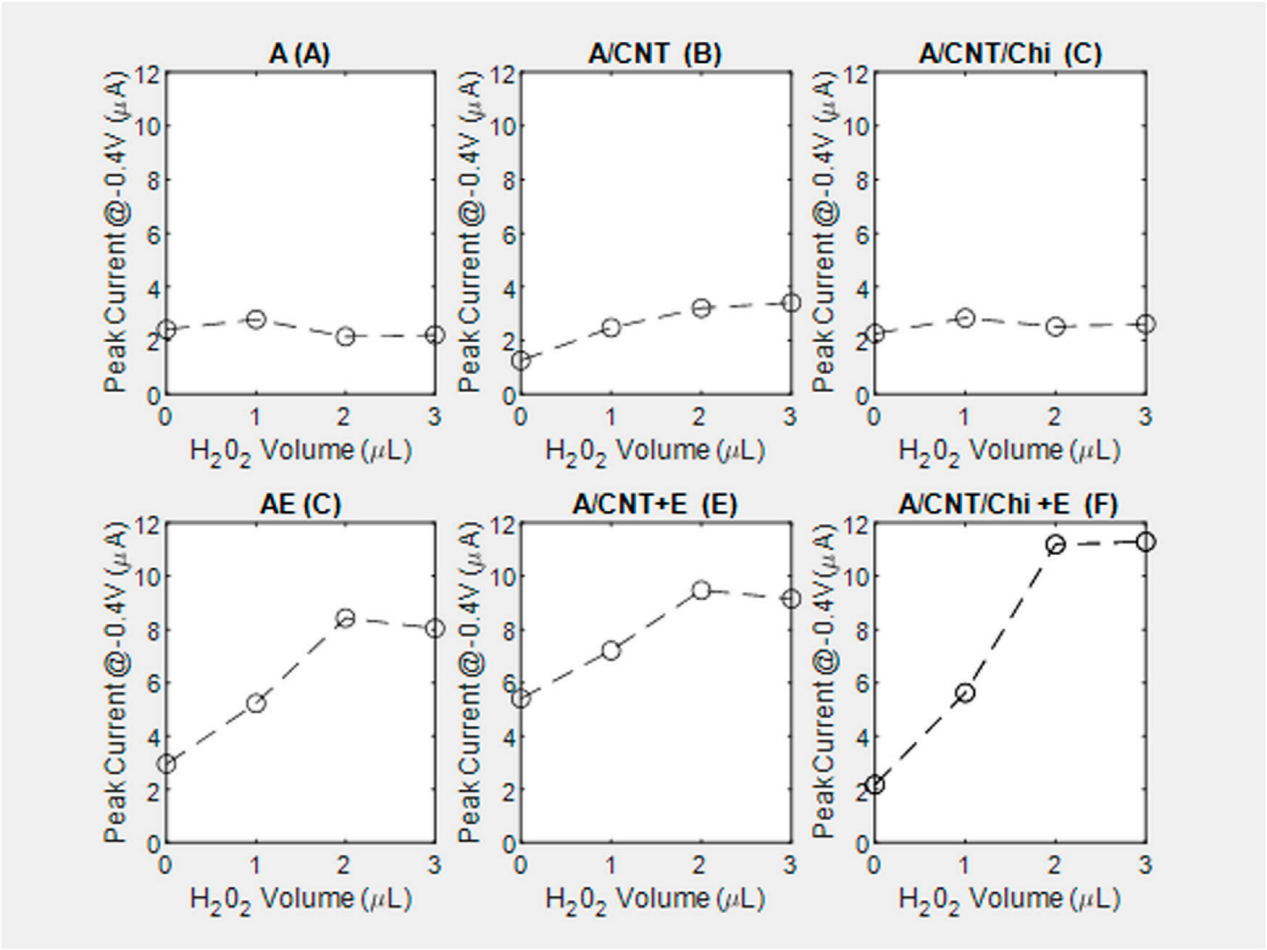
Peak current at −0.4 V relative to the background signal from the voltagrams of nanocomposite biosensing films, performed with a voltage sweep between 0.5 V and −0.8 V at a rate of 100 mV/s on controls and corresponding reactive catalase immobilized slabs in the top and bottom rows, respectively. Main plots capture substrate ranging from (0–3 µL) while insets contrast the overlay between (0and3 *µL*): (A1) alginate slabs **(A)**, (A2, inset); **(B)** alginate slabs with CNTs (A/CNT), (B2, inset); **(C)** alginate slabs with CNTs and chitosan (A/CNT/Chi), (C2,inset); **(D)** alginate slabs with CAT (AE), (D2,inset); **(E)** alginate slabs with CNTs and CAT (A/CNT + E), (E2, inset); **(F)** alginate slabs with CNTs, chitosan and CAT (A/CNT/Chi + E), (F2,inset).

**FIGURE 9:**
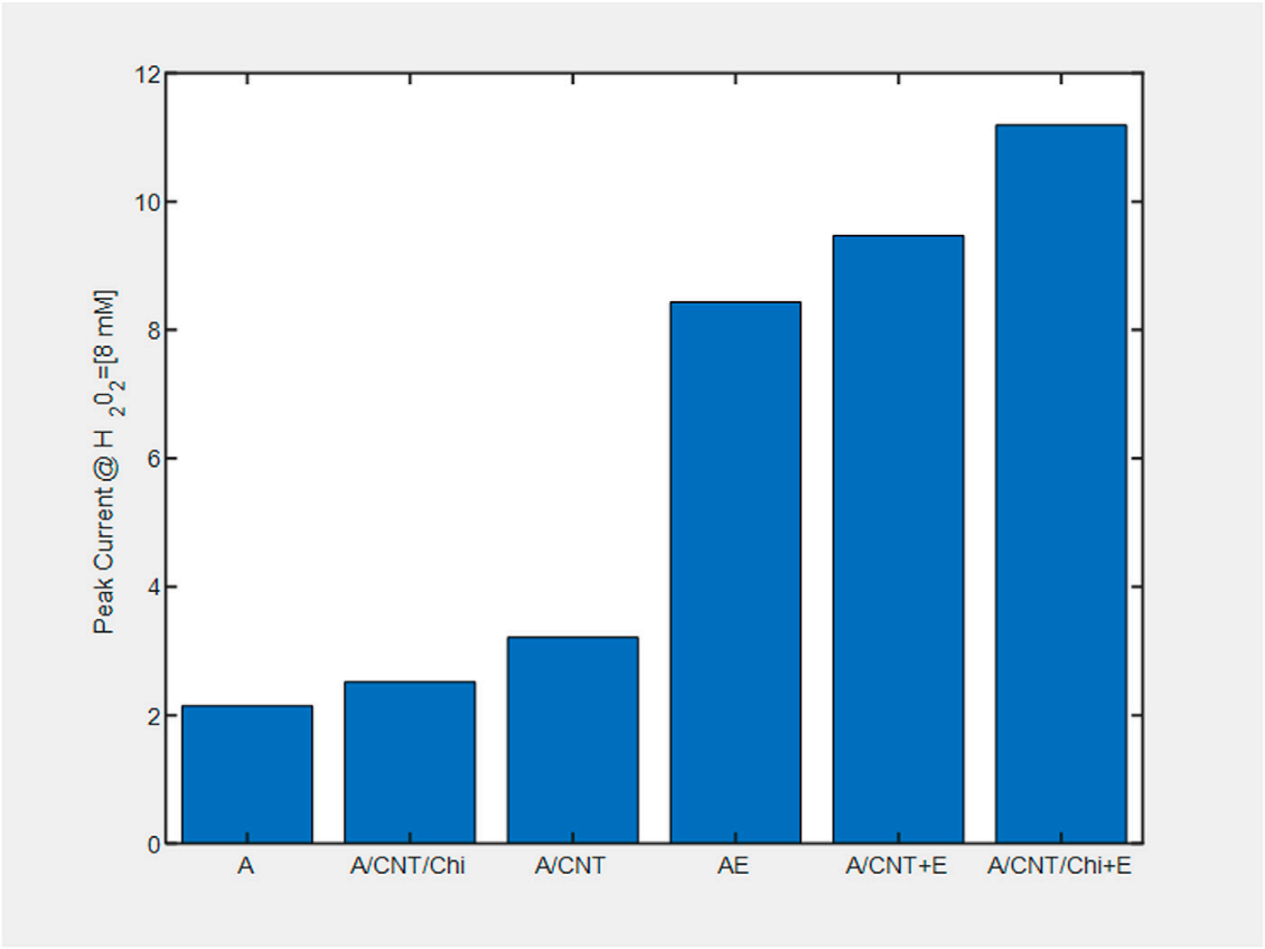
Peak current at −0.4 V relative to the background signal from the voltammograms of nanocomposite biosensing films for 2 μl of H_2_O_2_ (8 mM).

The pair of redox current peaks observed for each modified sensing platform were measured in the absence and presence of catalase. Specifically, from these voltammetric measurements (Figure 7), the peak current was identified relative to the background currents and translated into current vs substrate volume Figure 8. For all nanocomposite platforms, the peak current increased from 2.15 *μA* (A) to 8.43 μA (AE), from 3.21 μA (A/CNT) to 9.47 μA (A/CNT + E), and from 2.52 μA (A/CNT/Chi) to 11.19 μA (A/CNT/Chi + E = SCAT) respectively (Figure 8). All of these current values were taken with 2 μL of H_2_O_2_ added for comparison, which corresponds to a final concentration of 8 mM as the current values typically saturate at this concentration. In all three cases, CAT-immobilized nanocomposite platform resulted in a higher current value of the H_2_O_2_ reduction peak with respect to the bare nanocomposite platform. Such responses show the enhanced catalytic activity from the immobilized enzyme in agreement the amperometric study findings. As suggested by evidence, nanomaterial modified sensors (Figures 8E,F) are superior to nanomaterial-free sensors (AE) in terms of conductivity. Specifically, CNTs have the large surface area and porosity interacting with CAT for enhanced electron transfer as proven by literature (Salimi et al., 2005; Chen et al., 2007; Huang et al., 2011; Woo et al., 2012; Hong et al., 2013; Soto et al., 2021; Ahmad et al., 2022). Among various nanocomposites, alginate with CNT and chitosan (SCAT) showed the highest peak current in agreement with the amperometric measurements. Peak currents at −0.4 V for 2 μL of H_2_O_2_ (8 mM) are presented in Figure 9.

The conductivity of alginate hydrogels without any fillers can modulated from 0–1 mS/m with the upper end representing superionic capacitors as a function of the crosslinking state, divalent cross-linker ion, ionic strength of the medium and temperature (Esch et al., 1999; Khairou and Hassan, 2002; Kaklamani et al., 2018; Ji et al., 2022). In the current study, mediator-less conductance measurements enabling direct comparisons with the above-mentioned were not made. Graphene oxide (GO), reduced graphene oxide (RGO) and MWCNT have been used to modulate hydrogel conductivity (Golafshan et al., 2017; Liu et al., 2018; Serrano-Aroca et al., 2018; Fu et al., 2019; Li et al., 2019; McNamara et al., 2020; Raslan et al., 2021). While RGO and MWCNT are hydrophobic and GO is hydrophilic, all nanomaterials have exhibited the same level of teratogenicity in zebrafish (Liu et al., 2014; Shu et al., 2020). Relevant cyclic voltammetry studies range from optimal design of fiber-based capacitors the volumetric capacitance of MWCNT was approximately quadrupled for an RGO/MWCNT mixture (4 F cm^−3^) (Raslan et al., 2021) to mediated enzymatic reactions where chitosan and graphene were blended to measure metabolic activity (Feng and Wang, 2021). However, neither of the studies above emulate the nanocomposite stacking, diffusion limitations and the potassium hexacyanoferrate (II)/(III) complexes mediated conditions used in the current study.

In the absence of reaction (Figure 8, peak current increases with the addition of the MWCNT (A/CNT) to the alginate slab (A) confirming that MWCNT increases gel conductivity. The current reverts back to the baseline (A) with the physical adsorption of the polycationic chitosan (A/CNT/Chi). With regards to the kinetic mechanisms (Figures 8A,B), there is distinct difference between the peak currents observed in the absence of substrate (0 μL) with (5.41 μA) and without (2.96 μA) the MWCNT incorporation (Figures 7D,F) suggesting enzyme adsorption onto nanotubes via π-π interactions detected by CV studies of proteins on RGO [ 71] and electrochemical impedance spectroscopy (Chen et al., 2018). This change in formulation has also resulted in the flattening of the voltammogram peaks at −0.4 V. Chitosan incorporation has restored the shape of the voltammogram to that of the alginate enzyme (Figure 7F vs. Figure 7D) as well as the zero-substrate concentration peak current. Conductivity for samples with extremely low CNT loading values, which present no connectivity or close proximity between CNT bundles as is the case for the nanocomposites under observation (Figure 2B), showing an electrical conductivity characterized by a current/voltage dependence (Figure 8B) has been demonstrated for resistive polymer matrices (Earp et al., 2019). Since the electrical percolation limit for MWCNT immobilized in alginate has not been documented, future experiments will entail varying the nanofiller concentration to elucidate the conductive mechanism of MWCNT with the nanocomposite slabs.

#### Effect of H_2_O_2_ concentrations in the electrochemical response

Typically, the peak current values measured from nanocomposite electrodes without catalase remained constant with increasing concentrations of H_2_O_2_ (Figures 8A–C). In the presence of catalase, the peak current values initially increased with increasing concentrations of H_2_O_2_ and eventually saturated with 2 μL of H_2_O_2_ added, which corresponds to a final concentration of 8 mM of H_2_O_2_ (Figures 8D–F). It could be hypothesized that the zero-order kinetic behavior observed is due the background signal subtraction absent in the case of amperometry measurements.

The appearance of the anodic peak at −0.4 *V* in the presence of substrate is illustrated in Figures 7D,F.

Among different nanocomposite electrodes, the catalase immobilized sensor on alginate with CNTs and chitosan showed the highest current increase (1.13 μA/mM) as compared to the other formulations. The comparatively lower current increase for A/CNT + E (0.51 μA/mM) electrode was attributed to the higher current value in the absence of H_2_O_2_ as reflected by a baseline value of 5.41 μA (Figure 8F).

#### Effect of SPCE stability on the measurements

A single SPCE was used to conduct the voltammetry measurements in order to reduce the effect of sensor to sensor variability. The use of the DropSens identical to the current study for oxygen detection in room-temperature ionic liquids has been reported (Murugappan et al., 2011; Junqiao et al., 2017). In both cases, the C-SPEs were far inferior (i.e., higher LODs, large capacitive currents, more signal deterioration) compared to their Pt counterparts. In a parallel study, the root cause of the deterioration was investigated in the reverse reaction where reduction of oxygen was detected by voltammetry associated with the formation of H_2_O_2_ and water (Nissim and Compton, 2013). However, an additional signal was seen on the carbon paste electrode attributed to the initial formation of the superoxide radical anion, O_2_˙^‐^, suggesting that the predominant source of oxygen for this reaction was that dissolved in the carbon paste material rather than the aqueous solution.

Although the reduction of H_2_O_2_ is reversible, no additional peak was detected in the voltammograms associated with superoxide generation (Figures 8D,E). In the event that equilibrium shifts towards the reversible reaction, the hydrogel nanocomposite slab on the surface of which reaction occurs shields the SPCE from superoxide diffusion into the carbon paste. Replication is needed to ascertain this hypothesis but preliminary inspection of the SPCE sensor shown in Figure 2D support the above-stated hypotheses.

## 4 Conclusion

In this study, an electrochemical H_2_O_2_ biosensor based on screen printed carbon electrodes was developed by employing co-encapsulated catalase and multi-walled carbon nanotubes in alginate films coated with chitosan. Conditions for the fabrication and geometry of the sensors were optimized, and the thin films were systematically investigated for diffusive and electrical properties. The optimal sensor design demonstrated desirable traits such as high sensitivity (4.55 μA/mM) comparable to parallel detection CNT-based nano-environments, facile fabrication by polyelectrolyte complexation, and is made of proven biocompatible materials. The proposed sensing platform has a potential to be further developed as a third-generation biosensor where it promotes direct electron transfer without the need for an electron transfer mediator to be verified by cyclic voltammetry and electrochemical impedance spectroscopy. After determination of the electrical percolation limits using MWCNTs incorporation of other nanofillers namely transition metal dichalcogenides with proven sub *nM* H_2_O_2_ detection limits in cancer cells (Dou et al., 2018) will be investigated to improve the LOD. Future development may integrate microneedle arrays with the current sensing platform to detect hydrogen peroxide, glucose, or lactate in the subcutaneous tissue towards continuous health status monitoring under simulated physiological conditions.

## Data Availability

The original contributions presented in the study are included in the article/Supplementary Material, further inquiries can be directed to the corresponding authors.
